# Comparison of acute kidney injury between open and laparoscopic liver resection: Propensity score analysis

**DOI:** 10.1371/journal.pone.0186336

**Published:** 2017-10-13

**Authors:** Young-Jin Moon, In-Gu Jun, Ki-Hun Kim, Seon-Ok Kim, Jun-Gol Song, Gyu-Sam Hwang

**Affiliations:** 1 Department of Anesthesiology and Pain Medicine, Asan Medical Center, University of Ulsan College of Medicine, Seoul, Korea; 2 Department of Surgery, Asan Medical Center, University of Ulsan College of Medicine, Seoul, Korea; 3 Department of Clinical Epidemiology and Biostatistics, Asan Medical Center, University of Ulsan College of Medicine, Seoul, Korea; University of Sao Paulo Medical School, BRAZIL

## Abstract

The inflammatory response has been shown to be a major contributor to acute kidney injury. Considering that laparoscopic surgery is beneficial in reducing the inflammatory response, we compared the incidence of postoperative acute kidney injury between laparoscopic liver resection and open liver resection. Among 1173 patients who underwent liver resection surgery, 222 of 926 patients who underwent open liver resection were matched with 222 of 247 patients who underwent laparoscopic liver resection, by using propensity score analysis. The incidence of postoperative acute kidney injury assessed according to the creatinine criteria of the Kidney Disease: Improving Global Outcomes definition was compared between those 1:1 matched groups. A total 77 (6.6%) cases of postoperative acute kidney injury occurred. Before matching, the incidence of acute kidney injury after laparoscopic liver resection was significantly lower than that after open liver resection [1.6% (4/247) vs. 7.9% (73/926), *P* < 0.001]. After 1:1 matching, the incidence of postoperative acute kidney injury was still significantly lower after laparoscopic liver resection than after open liver resection [1.8% (4/222) vs. 6.3% (14/222), *P* = 0.008; odds ratio 0.273, 95% confidence interval 0.088–0.842, *P* = 0.024]. The postoperative inflammatory marker was also lower in laparoscopic liver resection than in open liver resection in matched set data (white blood cell count 12.7 ± 4.0 × 10^3^/μL vs. 14.9 ± 3.9 × 10^3^/μL, *P* < 0.001). Our findings suggest that the laparoscopic technique, by decreasing the inflammatory response, may reduce the occurrence of postoperative acute kidney injury during liver resection surgery.

## Introduction

As one of the most complex major abdominal surgeries, laparoscopic liver resection (LLR) is arguably the last frontier in minimally invasive surgery. Recently, owing to advances in surgical techniques, the use of LLR has been increasing rapidly [1]. Although several studies have shown the equality in outcomes between LLR and open liver resection (OLR), those studies were mainly focused on the surgical and oncological outcomes [2–6].

Postoperative acute kidney injury (AKI) is a serious postoperative complication with a detrimental impact on patient outcomes [7–9]. After liver resection surgeries, postoperative AKI has been associated with increased costs of care, morbidity, and mortality [10,11]. Although the exact mechanism of AKI is not yet fully understood, loss of homeostasis in the immune system and the ensuing inflammatory response are now believed to play major roles in the development of AKI [12–14]. Recent clinical studies found inflammatory markers to be independent predictors of AKI in patients with sepsis and after cardiovascular surgery [15,16].

One of the underestimated advantages of laparoscopic surgery is that it results in less immunologic modulation [17,18]. The immune-mediated inflammatory response is attenuated because of reduced surgical trauma and carbon dioxide pneumoperitoneum during laparoscopic surgery [19,20]. Considering that laparoscopic surgery is beneficial in reducing the inflammatory response, the laparoscopic technique may reduce the risk of postoperative AKI after liver resection. However, there is little information about the impact of LLR on postoperative AKI. We hypothesized that the laparoscopic technique during liver resection may exert a favorable effect on postoperative AKI. Thus, we aimed to compare the prevalence of postoperative AKI between OLR and LLR.

## Materials and methods

**Fig 1:**
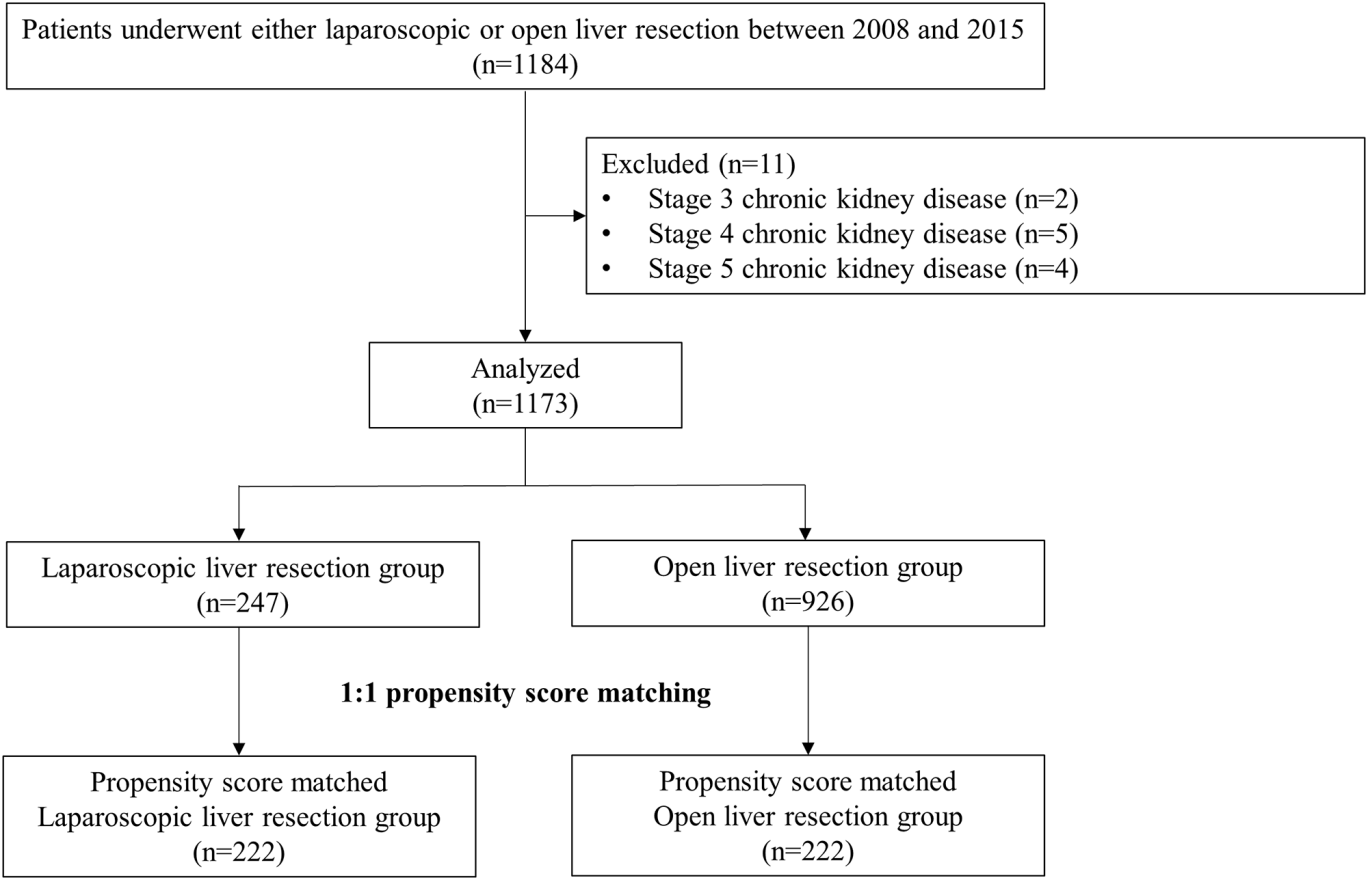
Study flow diagram.

After this retrospective observational study was approved by the institutional review board of Asan Medical Center, the data of all patients who underwent either OLR or LLR for primary hepatocellular carcinoma were reviewed. Informed consent was waived due to the retrospective nature of our study. LLR has been performed in Asan Medical Center since July 2007. Considering the learning curve of the surgeon, we included LLRs performed between January 2008 and October 2015. OLRs performed during the same period were also included. All surgeries were performed consecutively by a single surgeon (KHK). Of the 1184 identified patients, we excluded those with chronic kidney disease (CKD) of stage 3 or higher, as determined by the consulting nephrologists (n = 11) [21]. As the serum creatinine level of all patients was checked as part of the routine preoperative evaluation, we referred all patients with serum creatinine >1.5 mg/dL or those with any history of CKD to the consulting nephrologists for preoperative risk stratification. The final cohort comprised 1173 patients (Fig 1).

### Data collection

We collected the patients’ baseline characteristics, laboratory variables, and perioperative variables by using our institution’s patient record system. The baseline characteristics included patient age, sex, body mass index (BMI), model for end-stage liver disease (MELD) score, Child-Turcotte-Pugh grade, and comorbidities (hypertension, diabetes mellitus, and any history of ischemic heart disease, cerebrovascular disease, or heart failure). Variables related to the patients’ tumor characteristics included the largest tumor size and alpha-fetoprotein level. Laboratory data included hemoglobin, platelets, prothrombin time, serum creatinine, erum albumin, total bilirubin, white blood cell (WBC) count, aspartate transaminase, alanine transaminase, sodium, potassium, glucose, and total cholesterol. Intraoperative data included the extent of resection (major and minor), year of surgery, volume of infused fluids (crystalloid, synthetic colloid, and albumin), amount of transfusion, urine output, and operation time. Blood tests including serum creatinine, complete blood cell, and liver function tests were routinely conducted preoperatively as part of patient evaluation. Baseline laboratory values including kidney function were determined as the most recent measurements before the surgery.

### Anesthetic technique

General anesthesia was conducted with thiopental sodium 4–5 mg/kg, fentanyl 1–2 μg/kg, and rocuronium 0.6–1.2 mg/kg. Anesthesia was maintained with sevoflurane 2–4 vol% in 50% air/oxygen. Invasive arterial and central venous pressure monitoring were routinely conducted. Crystalloids (Ringer’s lactate or balanced isotonic electrolyte solution) and colloids [5% albumin or 6% hydroxyl ethyl starch 130/0.4 (Voluven^®^; Fresenius Kabi, Bad Homburg, Germany)] were infused depending on individual practitioner preferences. The total volume of hydroxyl ethyl starch did not exceed 20 mL/kg. Packed red blood cells were transfused when the patient’s hemoglobin was <8 mg/dL. For patients with a history of ischemic heart disease, hemoglobin levels were maintained at >10 mg/dL. Central venous pressure was maintained at <5 mmHg. If the mean arterial blood pressure was <65 mmHg, vasoactive drugs (phenylephrine, ephedrine, or norepinephrine) were administered.

### Surgical technique

Basic operative criteria were applied for LLR and OLR; however, LLR was offered to patients with smaller lesions (<5 cm). The surgical team allowed the patients to choose the surgical technique after receiving a comprehensive explanation about both LLR and OLR [22]. Liver resection was described according to Couinaud’s classification. Minor resection was defined when hepatic resection was limited to two or fewer segments, and the others were defined as a major resection [23]. Right anterior and posterior sectionectomy were classified as major resection, as advanced techniques and a longer operation time are frequently required for those types of hepatic resections [24]. For OLR, the abdomen was explored through a J-shaped incision. The hepatic parenchyma was divided by using an ultrasonic aspirator (CUSA; Valleylab, Boulder, CO, USA). After parenchymal transection, the hepatic vein was cut with either a vascular stapler or vascular clamps, and then sutured. For LLR, carbon dioxide was infused to keep the pneumoperitoneum pressure at <12 mmHg. Maximal muscle relaxation was achieved for an optimal surgical view throughout the procedure. After parenchymal dissection, the hepatic vein was ligated with an endoscopic stapler. The resected specimen was retrieved through a Pfannenstiel incision. Blood test measurements including serum creatinine, complete blood cell test, and liver function tests were routinely conducted on postoperative days 1, 2, 3, 5, and 7.

### Definition of outcomes and associated comorbidities

The primary endpoint was the development of AKI as defined according to the serum creatinine criteria of the Kidney Disease: Improving Global Outcomes (KDIGO) classification. Postoperative AKI was defined as a serum creatinine increase of ≥0.3 mg/dL within 2 days after surgery or a serum creatinine increase of ≥1.5 times within 7 days after surgery [25]. Other postoperative outcome variables, including maximum WBC count, development of stage 3 or higher CKD, renal replacement therapy, intensive care unit (ICU) admission, length of hospital stay, and mortality were evaluated in association with LLR. CKD was defined when the estimated glomerular filtration rate decreased to <60 mL·min^-1^·1.73 m^-2^ within a year after surgery on the second of two consecutive occasions at least 3 months apart [21]. The stages of CKD were classified according to the lowest estimated glomerular filtration ratio: stage 3, 30–59 mL·min^-1^·1.73 m^-2^; stage 4, 15–29 mL·min^-1^·1.73 m^-2^; and stage 5, <15 mL·min^-1^·1.73 m^-2^ or the need for dialysis.

### Statistical analysis

Parameters are presented as numbers and percentages, means ± standard deviation, or median with the interquartile range, as appropriate. Between-group comparisons were performed by using the chi-square test or Fisher’s exact test, and Student’s *t*-test or Mann-Whitney *U*-test, as appropriate. Independent risk factors for the occurrence of postoperative AKI were identified through multivariable logistic regression with backward elimination.

A 1:1 propensity score matching analysis was done to minimize the potential for bias in an observational study [26]. Propensity scores were estimated with the type of surgery as a dependent variable in multiple logistic regression analysis. All patients’ perioperative variables shown in Table 1 were included for propensity score generation. Missing values were imputed by using the Markov chain Monte Carlo method. Model discrimination was measured with *c* statistics (0.700). Model calibration was performed with Hosmer-Lemeshow statistics (χ2 = 5.9042, *df* = 8, *P* = 0.658). Propensity score matching was performed through greedy matching by using a caliper of 0.2 standard deviations of the logit of the propensity score. The absolute standardized differences were used to diagnose the balance after matching, and all standardized differences were <0.1. Moreover, comparisons between the two groups were performed with the paired t-test or Wilcoxon signed-rank test for continuous variables and with the McNemar test for categorical variables. Cox regression analysis was used to compare the survival rate between patients with and without postoperative AKI in both before and after matched data set. *P* values <0.05 were considered statistically significant. Statistical analyses were done with SAS^®^ version 9.3 (SAS Institute Inc., Cary, NC, USA).

**Table 1:** Patient demographics and perioperative variables before and after matching.

		Before Matching			After Matching			
	Total (N = 1173)	Laparoscopic liver resection (n = 247)	Open liver resection (n = 926)	*P* value	Laparoscopic liver resection (n = 222)	Open liver resection (n = 222)	*P* value	Standardized difference
**Demographics**								
Age (years)	55.7 ± 10.2	54.9 ± 10.4	55.9 ± 10.2	0.102	55.5 ± 10.2	54.7 ± 10.3	0.405	0.080
Sex, male	951 (81.1%)	173 (70.0%)	778 (84.0%)	<0.001	162 (73.0%)	167 (75.2%)	0.300	0.049
Body mass index (kg/m^2^)	24.2 ± 2.9	24.2 ± 2.8	24.2 ± 2.9	0.710	24.4 ± 2.7	24.6 ± 2.9	0.414	0.077
MELD score	7.0 [6.0–8.0]	7.0 [6.0–8.0]	7.0 [6.0–8.0]	0.198	7.0 [6.0–8.0]	7.0 [6.0–8.0]	0.970	0.019
Child-Turcotte-Pugh grade				1.000			1.000	0.004
A	1155 (98.5%)	243 (98.4%)	912 (98.5%)		221 (99.9%)	222 (100%)		
B	18 (1.5%)	4 (1.6%)	14 (1.5%)		1 (0.5%)	0 (0.0%)		
Diabetes	75 (6.4%)	13 (5.3%)	62 (6.7%)	0.414	12 (5.4%)	13 (5.9%)	0.841	0.020
Hypertension	80 (6.8%)	15 (6.1%)	65 (7.0%)	0.600	14 (6.3%)	13 (5.9%)	0.841	0.019
Ischemic heart disease	9 (0.8%)	1 (0.4%)	8 (0.9%)	0.694	1 (0.5%)	1 (0.5%)	1.000	<0.001
Cerebrovascular disease	4 (0.3%)	1 (0.4%)	3 (0.3%)	1.000	1 (0.5%)	2 (0.9%)	0.564	0.071
Tumor size (cm)	4.6 ± 3.9	2.8 ± 1.3	5.1 ± 4.1	<0.001	3.3 ± 1.4	3.4 ± 1.8	0.714	0.035
Alpha-fetoprotein (ng/mL)	12.6 [3.9–170.0]	8.9 [3.1–66.7]	14.6 [4.2–249.0]	0.022	12.6 [3.6–224.0]	11.1 [3.8–254.0]	0.825	0.028
**Operation type**								
Year of surgery (years from 2008)	4 [2–6]	5 [3–6]	4 [2–5]	<0.001	5 [3–6]	5 [3–7]	0.236	0.077
Major	790 (67.3%)	100 (40.5%)	690 (74.5%)	<0.001	100 (45.1%)	103 (46.4%)	0.758	0.028
Minor	383 (32.7%)	147 (59.5%)	236 (25.5%)		122 (55.0%)	119 (53.6%)		
Right lobectomy	227 (19.4%)	39 (15.8%)	188 (20.3%)		39 (17.6%)	26 (11.7%)		
Extended right lobectomy	14 (1.2%)	0 (0.0%)	14 (1.5%)		0 (0.0%)	3 (1.4%)		
Right posterior sectionectomy	194 (16.5%)	19 (7.7%)	175 (18.9%)		19 (8.6%)	33 (14.9%)		
Right anterior sectionectomy	183 (15.6%)	7 (2.8%)	176 (19.0%)		7 (3.2%)	28 (12.6%)		
Left lobectomy	140 (11.9%)	35 (14.2%)	105 (11.3%)		35 (15.8%)	9 (4.1%)		
Extended left lobectomy	32 (2.7%)	0 (0.0%)	32 (3.5%)		0 (0.0%)	4 (1.8%)		
Left lateral sectionectomy	93 (7.9%)	71 (28.7%)	22 (2.4%)		63 (28.4%)	12 (5.4%)		
Bisegmentectomy	67 (5.7%)	3 (1.2%)	64 (6.9%)		2 (0.9%)	31 (14.0%)		
Monosegmentectomy	60 (5.1%)	9 (3.6%)	51 (5.5%)		5 (2.3%)	28 (12.6%)		
Partial hepatectomy	163 (13.9%)	64 (25.9%)	99 (10.7%)		52 (23.4%)	48 (21.6%)		
**Preoperative variables**								
Creatinine (mg/dL)	0.8 ± 0.2	0.8 ± 0.2	0.8 ± 0.2	0.153	0.8 ± 0.2	0.8 ± 0.2	0.919	0.011
eGFR ≥ 90 mL·min^-1^·1.73 m^-2^	307 (26.2%)	92 (37.2%)	215 (23.2%)		81 (36.5%)	74 (33.3%)		
eGFR 60–89 mL·min^-1^·1.73 m^-2^	866 (73.8%)	155 (62.8%)	711 (78.8%)		141 (63.5%)	148 (66.7%)		
White blood cell count (×10^3^/μL)	5.4 ± 1.8	5.14 ± 1.64	5.49 ± 1.80	0.006	5.2 ± 1.7	5.1 ± 1.5	0.714	0.035
Hemoglobin (mg/dL)	13.9 ± 1.6	13.89 ± 1.62	13.95 ± 1.58	0.594	13.9 ± 1.6	13.9 ± 1.6	0.921	0.009
Platelets (×10^3^/μL)	163.9 ± 66.8	152.8 ± 52.7	166.9 ± 69.8	0.010	151.8 ± 53.2	150.9 ± 56.9	0.860	0.017
Prothrombin time (INR)	1.04 ± 0.08	1.04 ± 0.08	1.04 ± 0.08	0.304	1.0 ± 0.1	1.0 ± 0.1	0.691	0.036
Albumin (g/dL)	3.8 ± 0.4	3.8 ± 0.4	3.8 ± 0.4	0.134	3.8 ± 0.4	3.9 ± 0.4	0.709	0.036
Total bilirubin (mg/dL)	0.8 ± 0.4	0.8 ± 0.4	0.8 ± 0.4	0.005	0.8 ± 0.4	0.8 ± 0.3	0.455	0.047
Aspartate transaminase (IU/L)	39.8 ± 30.6	31.8 ± 14.3	41.9 ± 33.4	<0.001	32.6 ± 14.8	34.1 ± 17.4	0.283	0.102
Alanine transaminase (IU/L)	37.1 ± 28.1	31.9 ± 22.2	38.4 ± 29.4	<0.001	33.0 ± 23.0	34.3 ± 21.6	0.350	0.059
Sodium (mmol/L)	139.8 ± 2.6	140.3 ± 2.3	139.7 ± 2.6	0.002	140.2 ± 2.4	140.3 ± 2.4	0.715	0.035
Potassium (mmol/L)	4.2 ± 0.3	4.2 ± 0.3	4.2 ± 0.4	0.992	4.2 ± 0.3	4.2 ± 0.3	0.929	0.014
Chloride (mmol/L)	103.8 ± 2.8	104.1 ± 2.5	103.7 ± 2.9	0.215	104.0 ± 2.6	104.2 ± 2.5	0.574	0.053
Glucose (mg/dL)	118.5 ± 47.2	116.8 ± 49.8	118.9 ± 46.5	0.186	118.4 ± 51.5	117.9 ± 51.7	0.969	0.011
Total cholesterol (mg/dL)	163.5 ± 35.2	161.4 ± 31.4	164.0 ± 36.2	0.252	161.8 ± 31.1	163.2 ± 31.4	0.625	0.046
**Intraoperative variables**								
Crystalloid (mL)	2263.5 ± 983.3	2164.3 ± 994.1	2290 ± 979.2	0.074	2230.5 ± 1014.0	2171.2 ± 892.3	0.676	0.043
Synthetic colloid (mL)	573 ± 320.9	523.7 ± 283.5	581.1 ± 326.2	0.086	533.42 ± 299.0	491.6 ± 250.6	0.337	0.017
5% Albumin (mL)	583.7 ± 561.4	437.5 ± 210.2	630.2 ± 627.9	0.269	430.6 ± 221.1	447.4 ± 223.9	0.768	0.007
Incidence of transfusion (n)	76 (6.5%)	4 (1.2%)	72 (7.1%)	<0.001	4 (1.8%)	3 (1.4%)	1.000	0.007
Packed red blood cell (units)	3.6 ± 3.1	3.3 ± 1.2	3.6 ± 3.1	0.752	4.33 ± 1.1	2.0 ± 0.0	0.073	
Fresh frozen plasma (units)	3.1 ± 1.8	1.0 ± 0.0	3.3 ± 1.8	0.175	1.0 ± 0.0	2.0 ± 0.0		
Platelet concentration (units)	9.0 ± 3.8	15.0 ± 0.0	8.3 ± 3.2	0.064	15 ± 0.0	10 ± 0.0		
Urine output (mL)	507.5 ± 354.7	478.3 ± 362.2	515.3 ± 352.5	0.146	487.1 ± 370.9	430.5 ± 239.7	0.257	0.046
Duration of surgery (min)	271.1 ± 81.2	268.8 ± 93.3	271.7 ± 77.6	0.656	266.0 ± 94.0	253.1 ± 69.2	0.204	0.074

Values are expressed as the mean ± standard deviation, median [interquartile range], or n (%).

MELD, model for end-stage liver disease; eGFR, estimated glomerular filtration rate; INR, international normalized ratio.

## Results

### Analyses before matching

**Fig 2:**
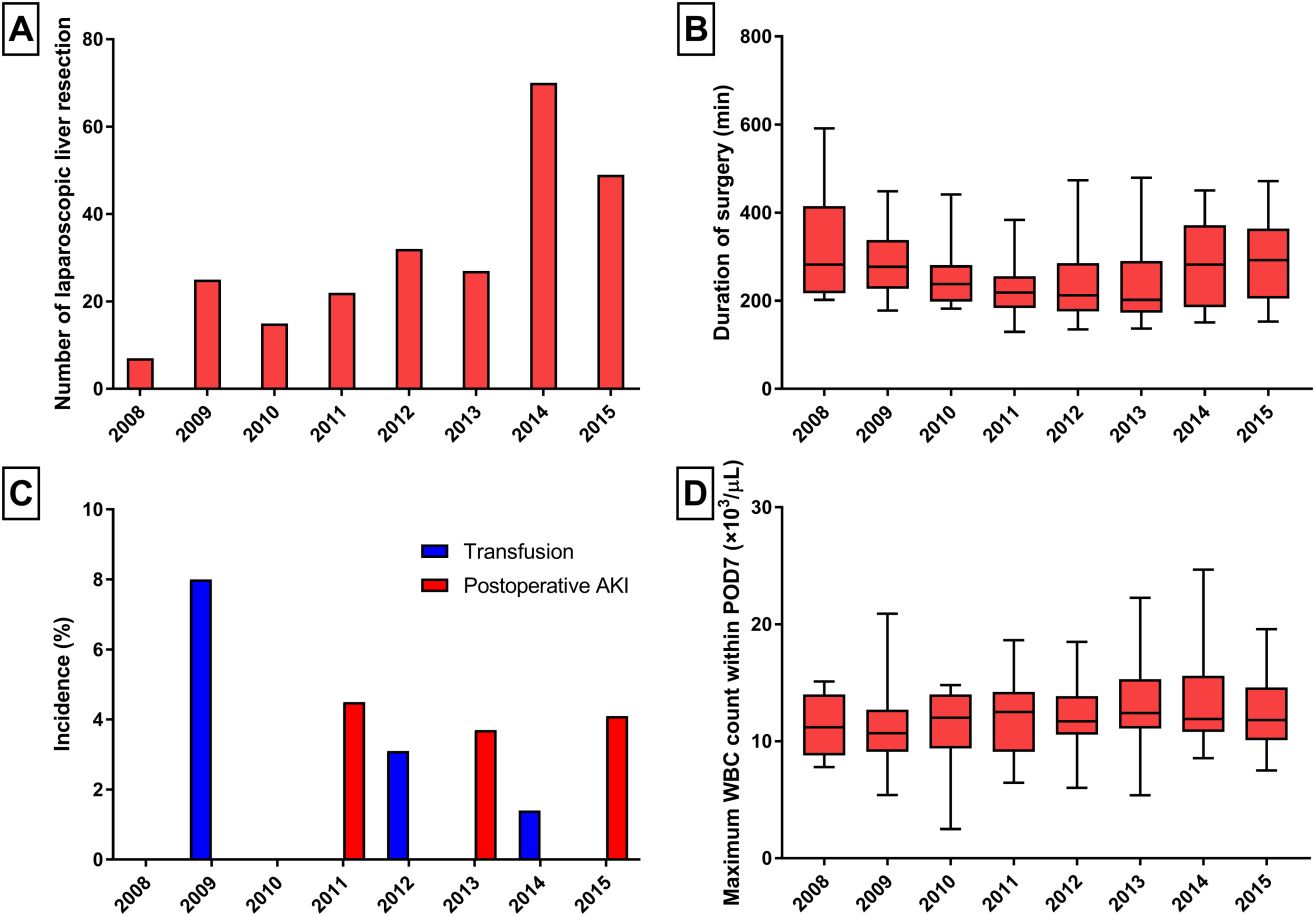
Time trend regarding laparoscopic liver resection. Year trends of (a) performed cases, (b) surgical duration, (c) transfusion, postoperative AKI, and (d) inflammatory marker.

A total of 1173 patients were enrolled in this study with a median follow-up period of 3.4 years (interquartile range 1.6–8.0 years). Among these patients, 247 underwent LLR and 926 underwent OLR for primary hepatocellular carcinoma. Major liver resections were performed in 40.5% (n = 100) of LLR cases and 74.5% (n = 690) of OLR cases. Regarding LLR, the time trend of the number of performed cases, surgical duration, incidence of transfusion and postoperative AKI, and inflammatory marker were shown in Fig 2.

Table 1 shows the preoperative and intraoperative variables of the two groups categorized according to the surgery type. Before matching, there was a significant heterogeneity between the two groups. The LLR group tended to have more female patients (*P* < 0.001), smaller tumor size (*P* < 0.001), lower alpha-fetoprotein (*P* = 0.022), more recent cases (*P* < 0.001), more minor resections (*P* < 0.001), lower WBC count (*P* = 0.006), lower platelets (*P* = 0.010), lower aspartate aminotransferase and alanine aminotransferase (*P* < 0.001 for both), lower total bilirubin (*P* = 0.005), higher sodium (*P* = 0.002), and lower incidence of transfusion (*P* < 0.001) than the OLR group.

**Fig 3:**
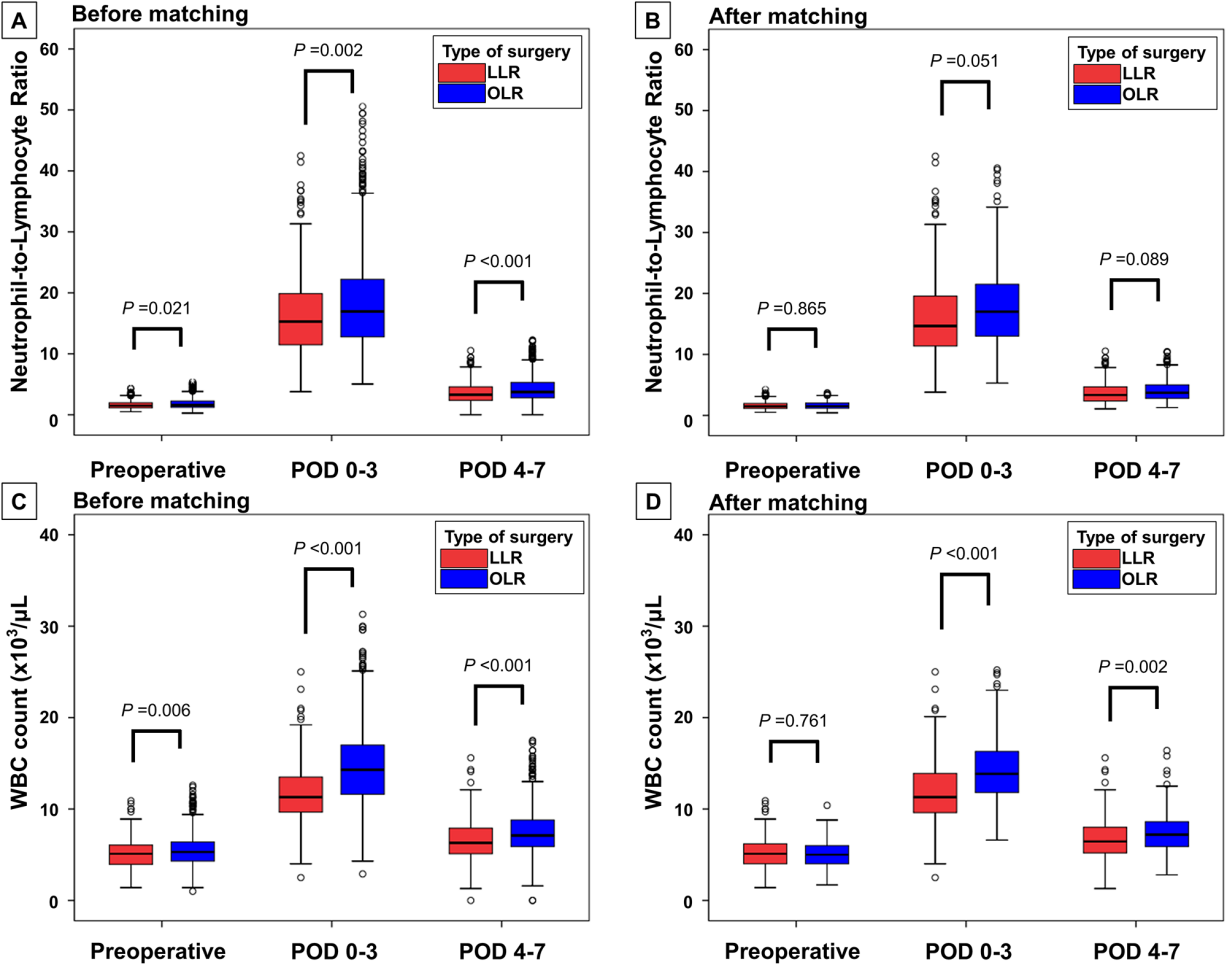
Comparison of perioperative inflammatory markers. Comparison of maximum neutrophil-to-lymphocyte ratio and white blood cell count within postoperative day 7 between laparoscopic and open liver resection (a, c) before and (b, d) after matching. In matched set data, white blood cell count was significantly lower in the LLR group during the first postoperative week. LLR, laparoscopic liver resection; OLR, open liver resection; WBC, white blood cell; POD, postoperative day.

**Fig 4:**
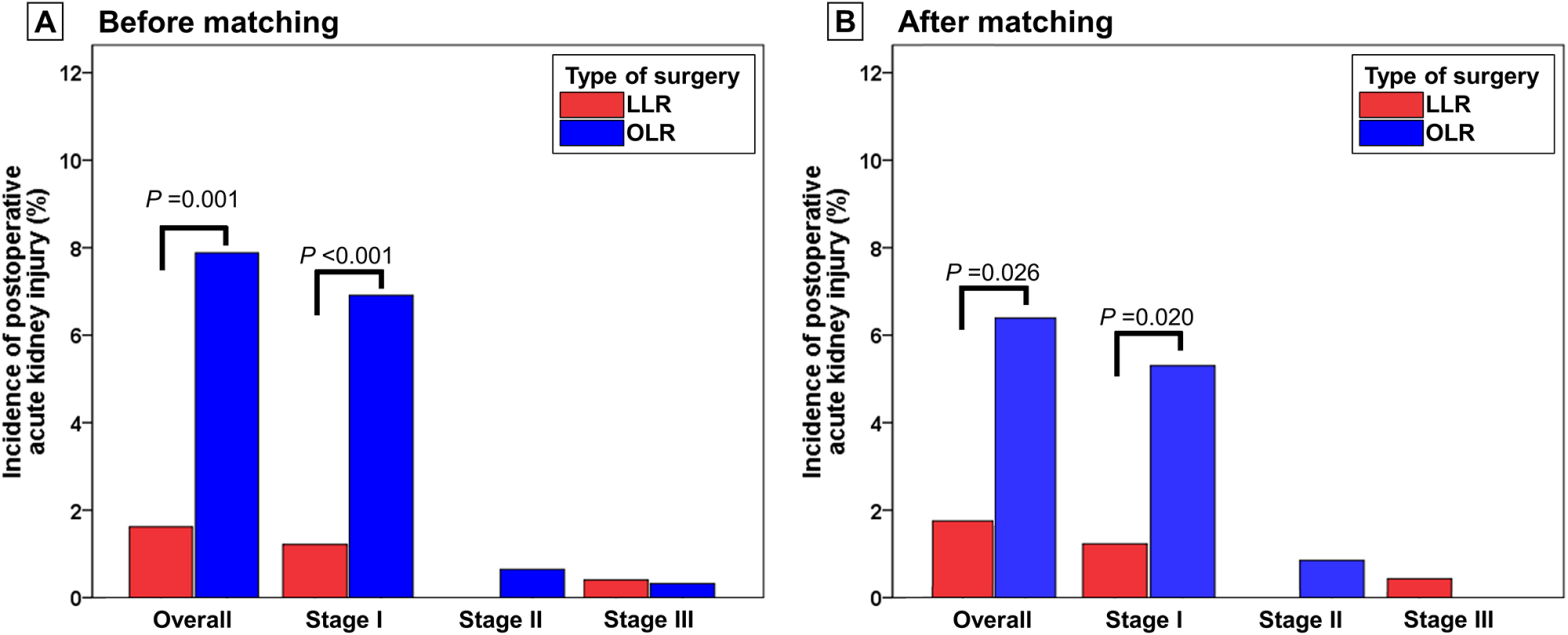
Comparison of postoperative acute kidney injury. The overall incidence of postoperative acute kidney injury was significantly lower after laparoscopic liver resection than after open liver resection. This result was consistent between (a) before and (b) after matching. LLR, laparoscopic liver resection; OLR, open liver resection.

Table 2 shows the postoperative inflammatory variables and postoperative outcomes of the two groups. Within postoperative day 7, the maximum neutrophil-to-lymphocyte ratio was significantly lower in the LLR group than in the OLR group (16.8 ± 7.9 vs. 18.6 ± 8.9, *P* = 0.004; Fig 3A). The maximum WBC count was significantly lower in the LLR group than in the OLR group (12.6 ± 3.9 vs. 15.4 ± 4.6, *P* < 0.001; Fig 3C). Postoperative AKI occurred less frequently after LLR than after OLR [4/247 (1.6%) vs. 73/926 (7.9%), *P* = 0.001; Table 2]. As shown in Fig 4A, the specific incidence of stage 1, 2, and 3 postoperative AKI after LLR was 1.6% (3/247), 0.0% (0/247), and 0.4% (1/247), respectively. The specific incidence of stage 1, 2, and 3 postoperative AKI after OLR was 6.9% (64/926), 0.6% (6/926), and 0.3% (3/926), respectively.

**Table 2:** Comparison of inflammatory markers, kidney outcome, and hospital course.

	Before Matching			After Matching		
	Laparoscopic liver resection (n = 247)	Open liver resection (n = 926)	*P* value	Laparoscopic liver resection (n = 222)	Open liver resection (n = 222)	*P* value
**Postoperative inflammatory variables**						
Maximum neutrophil-to-lymphocyte ratio within POD 7	16.8 ± 7.9	18.6 ± 8.9	0.004	16.6 ± 7.8	17.9 ± 6.7	0.067
Maximum WBC count within POD 7 (×10^3^/μL)	12.6 ± 3.9	15.4 ± 4.6	<0.001	12.7 ± 4.0	14.9 ± 3.9	<0.001
**Postoperative serum creatinine (mg/dL)**						
Maximal serum creatinine within POD 7	0.85 ± 0.27	0.92 ± 0.26	0.001	0.87 ± 0.27	0.89 ± 0.23	0.286
Serum creatinine at POD 90	0.81 ± 0.20	0.82 ± 0.22	0.546	0.81 ± 0.20	0.79 ± 0.15	0.106
Serum creatinine at 1 year	0.83 ± 0.22	0.87 ± 0.31	0.041	0.84 ± 0.22	0.83 ± 0.21	0.643
**Postoperative outcomes**						
Acute kidney injury	4/247 (1.6%)	73/926 (7.9%)	0.001	4/222 (1.8%)	14/222 (6.3%)	0.026
KDIGO grade 1/2/3	3/0/1	64/6/3	0.004	3/0/1	12/2/0	0.035
Odds ratio for acute kidney injury (95% confidence interval)	0.192 (0.070–0.532)	1	0.001	0.273 (0.088–0.842)	1	0.024
Chronic kidney disease	4 (1.6%)	38 (4.1%)	0.094	4 (1.8%)	8 (3.6%)	0.380
Stage 3/4/5	1/1/2	24/3/11	0.188	1/1/2	7/1/0	0.088
Hospital stay	19 [14–25]	18 [14–24]	0.445	18.5 [14–25]	18 [14–24]	0.977
Admission to intensive care unit	17 (6.9%)	70 (7.6%)	0.718	16 (7.2%)	19 (8.7%)	0.602
Mortality	6 (2.4%)	42 (4.5%)	0.138	6 (2.7%)	5 (2.3%)	0.763

Values are expressed as mean ± standard deviation, median [interquartile range], or n (%).

POD, postoperative day; WBC, white blood cell; KDIGO, the Kidney Disease: Improving Global Outcomes.

In multivariable analysis, LLR was found to be an independent contributor to the lower occurrence of postoperative AKI [odds ratio (OR) 0.228; 95% confidence interval (CI) 0.082–0.635, *P* = 0.005] after adjustment for clinical covariates (Table 3). Higher BMI (OR 1.125; 95% CI 1.039–1.219, *P* = 0.004), albumin concentration (OR 0.453; 95% CI 0.253–0.812, *P* = 0.008), and transfusion amount (OR 2.125; 95% CI 1.028–4.392, *P* = 0.042) were also independently associated with the occurrence of postoperative AKI.

**Table 3:** Univariate and multivariate analyses for postoperative acute kidney injury.

	Univariate		Multivariate	
	Odds ratio (95% CI)	*P* value	Odds ratio (95% CI)	*P* value
Laparoscopic surgery	0.192 (0.070–0.532)	0.001	0.228 (0.082–0.635)	0.005
Male sex	2.093 (0.991–4.417)	0.053		
Body mass index	1.106 (1.022–1.197)	0.012	1.125 (1.039–1.219)	0.004
MELD score	1.164 (0.976–1.388)	0.092		
Diabetes mellitus	2.066 (0.987–4.322)	0.054	2.002 (0.936–4.283)	0.073
Serum albumin	0.415 (0.240–0.716)	0.002	0.453 (0.253–0.812)	0.008
Total bilirubin	1.721 (1.006–2.944)	0.048		
Transfusion	2.983 (1.495–5.952)	0.002	2.125 (1.028–4.392)	0.042

CI, confidence interval; MELD, model for end-stage liver disease.

Within a year from the surgery, CKD was diagnosed in 4 (1.6%) patients in the LLR group and in 38 (4.1%) patients in the OLR group (*P* = 0.094). The numbers of patients with CKD stage 3, 4, and 5 were 1 (0.5%), 1 (0.5%), and 2 (0.9%) after LLR and 24 (2.6%), 3 (0.3%), and 11 (1.2%) after OLR, respectively. ICU admission, length of hospital stay, and mortality were not statistically different between the LLR and OLR groups (all *P* > 0.05, Table 2).

### Analyses after matching

The postoperative inflammatory variables were significantly lower in the LLR group than in the OLR group even after matching (Table 2). The maximum neutrophil-to-lymphocyte ratio within postoperative day 7 was 16.6 ± 7.8 for the LLR group and 17.9 ± 6.7 for the OLR group (*P* = 0.067, Fig 3B). The maximum WBC count within postoperative day 7 was 12.7 ± 4.0 for the LLR group and 14.9 ± 3.9 for the OLR group (*P* < 0.001, Fig 3D).

The incidence of postoperative AKI after LLR was still significantly lower than that after OLR [4/222 (1.8%) vs. 14/222 (6.3%), *P* = 0.026; Table 2, Fig 4B]. The specific incidence of stage 1, 2, and 3 postoperative AKI after LLR was 1.4% (3/222), 0.0% (0/222), and 0.5% (1/222), respectively. The specific incidence of stage 1, 2, and 3 postoperative AKI after OLR was 5.4% (12/222), 0.9% (2/222), and 0.0% (0/222), respectively.

Postoperative outcomes including CKD, renal replacement therapy, hospital stay, ICU admission, and mortality were not significantly different between the two groups after matching (*P* > 0.100 for all).

**Fig 5:**
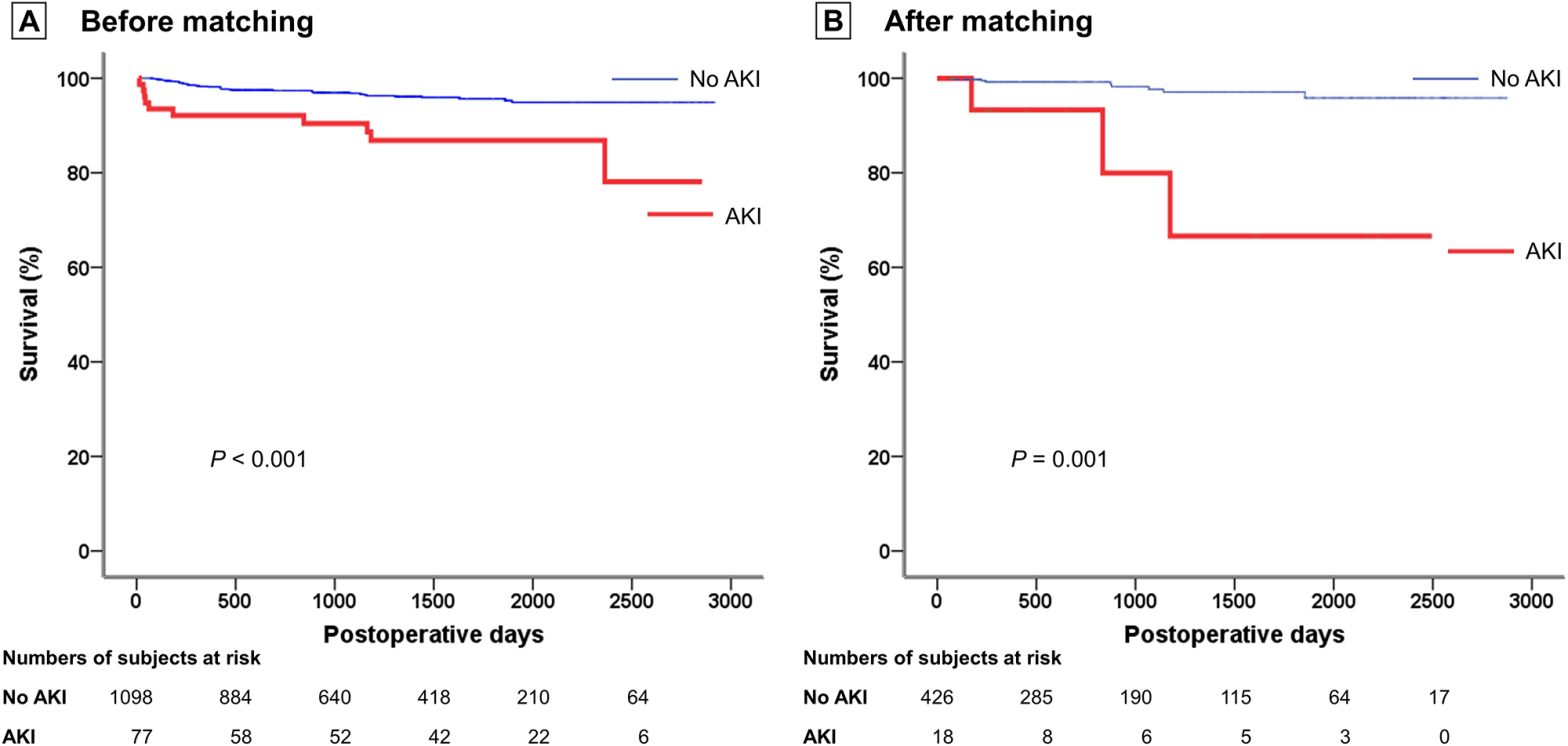
Survival curve according to the occurrence of postoperative AKI. Survival plot with Cox regression model demonstrated a significantly higher survival rate among patients with postoperative AKI (a) before (b) after matching. AKI, acute kidney injury.

Kaplan-Meier survival analysis showed a significant difference in mortality between the AKI and no AKI groups before and after matching (*P* < 0.001 for both, Fig 5).

## Discussion

In this observational study, the AKI incidence in LLR was 1.6%, which was significantly lower than that in OLR (7.9%). This result remained consistent after adjustment for important preoperative and intraoperative confounders with propensity score matching analysis. The significance of LLR was consistently found during multivariable analysis. In addition, there was a significant association between LLR and lower WBC count before and after matching.

To date, only a few studies have reported the exact incidence of AKI after LLR despite the recent increase in the number of LLR surgeries performed. Our results showed that the incidence of AKI after LLR was 1.6% according to the KDIGO criteria. In a previous literature review of a total of 2804 patients undergoing LLR, only one case of renal failure was reported [27]. However, most of the reviewed studies were not focused on postoperative renal function. Only a few studies actually described renal complications; however, their definitions were not clear. Recently, two case-matched studies reported that the incidences of renal complication after LLR were 1.1% (1/88) and 4.4% (2/45) [3,5] according to the Dindo-Clavien classification [28]. Although this classification system is widely used for most postoperative complications, it is not commonly used for evaluating postoperative AKI. In our study, we applied the KDIGO criteria, which have been validated to predict patients’ renal complications and other morbidities, and mortality [9,29].

Although the exact cause of AKI in patients with LLR has not been established, we presumed that the attenuation of the postoperative inflammatory response might have affected the occurrence of postoperative AKI after LLR. Previous studies have shown that laparoscopic surgery, by decreasing surgical trauma, induces less systemic inflammatory response than open surgery [30]. Some studies demonstrated that inflammatory markers such as WBC count, C-reactive protein level, and interleukin-6 level were significantly lower in laparoscopic surgery than in open surgery [31,32]. Notably, higher levels of postoperative inflammatory markers have been strongly linked to various organ injuries including postoperative AKI [33]. In the past decade, much knowledge has been generated about the pathophysiology of AKI and its association with the inflammatory response [12,13]. After the initial insult to the kidney, under ideal conditions, a balance between inflammatory and anti-inflammatory factors ensures robust tissue repair and restoration of homeostatic conditions. However, disruption of the balance in the immune system may hinder the normal repair process and lead to extensive kidney injury. The immunologic benefit of laparoscopic surgery may facilitate preventing the development of postoperative AKI. In accordance with previous reports [34–36], our study demonstrated that the WBC count was significantly lower in LLR than in OLR.

There are theoretical concerns that the laparoscopic technique may predispose patients to AKI during liver resection, because LLR is not only technically difficult to perform but also needs a long duration of pneumoperitoneum. Previous studies have shown that creatinine clearance and urine output were decreased during pneumoperitoneum [37]. In addition, owing to a potential bleeding risk during laparoscopic parenchymal dissection, the possibility of intraoperative hypotension and hypovolemia may render patients more susceptible to the development of postoperative AKI. However, some studies demonstrated that renal function would not be impaired if the volume status is adequate and the intra-abdominal pressure is maintained at about 10–15 mmHg during pneumoperitoneum [37,38]. As we vigorously attempted to maintain the patients’ hemodynamics and restricted intra-abdominal pressure at a low level of 12 mmHg during LLR, the patients might not be influenced by the potential adverse effects of pneumoperitoneum. Taken together, with proper intraoperative management, LLR may offer benefits related to kidney protection for patients.

Transfusion has been known to be one of the major contributors to postoperative AKI development. A recent study demonstrated that the maximum postoperative creatinine level of the LLR group was significantly lower than that of the OLR group (0.84 vs. 1.18, *P* < 0.001) [4], and other studies revealed that the incidence of AKI was lower in the laparoscopic surgery group, although it was not statistically significant [39,40]. The authors of the above studies suggested that lower AKI incidence may be attributed to the lower incidence of blood product transfusion after laparoscopic surgery. Our result confirmed the significance of transfusion on the occurrence of postoperative AKI. However, notably, even after adjustment for the bias of transfusion through propensity score matching and multivariable analysis, the laparoscopic technique was shown to still have the favorable effect of preventing the occurrence of postoperative AKI.

The current study also showed that diabetes, higher BMI, and lower postoperative albumin are risk factors for postoperative AKI. Diabetes is a well-known risk factor for AKI after various types of surgery including liver resection [11]. In terms of AKI, our current study showed the negative effect of higher BMI [41], although recent studies in obese patients undergoing LLR showed contradictory results with respect to morbidity [6,42]. Low serum albumin concentration has recently been associated with AKI after various types of surgery [41,43].

The association between postoperative AKI and long-term adverse renal outcomes including CKD has been demonstrated in previous studies [39,44]. The initiating mechanism and the subsequent maladaptive response after AKI have been suspected to damage the ability of the kidney to return to its normal function, thus increasing the probability of CKD [45]. In contrast to previous studies, our study showed that the incidence of postoperative AKI was reduced in the LLR group; however, its favorable effect did not result in any significant difference in the CKD rate. However, as our primary outcome was focused on the early postoperative period, long-term clinical confounders were not considered in the analysis. In addition, considering the low incidence of AKI in our study, the number of patients was not sufficient to draw reliable conclusions about its progression to CKD.

The current study has several limitations. First, as this study was retrospective in nature, we used propensity score matching to mitigate selection bias. However, not all confounding factors could be controlled. Second, because the current findings were from an observational analysis, we could not verify the causal relationship between LLR and its protective effect against postoperative AKI. Third, as this was a single-center study with all surgeries conducted by one experienced surgeon, caution should be exercised when interpreting the results of this study.

In conclusion, this propensity score-matched observational study showed that the incidence of postoperative AKI was lower after LLR than after OLR. The laparoscopic technique may play a protective role against the occurrence of postoperative AKI during liver resection surgery.

## Supporting information

S1 File Study’s underlying data set. We provide complete data set of our study after anonymization.
(XLSX)

## Abbreviations

AKI: acute kidney injury
CKD: chronic kidney disease
ICU: intensive care unit
KDIGO: Kidney Disease: Improving Global Outcomes
LLR: laparoscopic liver resection
MELD: model for end-stage liver disease
OLR: open liver resection
